# Differential replication of avian influenza H9N2 viruses in human alveolar epithelial A549 cells

**DOI:** 10.1186/1743-422X-7-71

**Published:** 2010-03-25

**Authors:** Davy CW Lee, Chris KP Mok, Anna HY Law, Malik Peiris, Allan SY Lau

**Affiliations:** 1 Cytokine Biology Group, Department of Paediatrics and Adolescent Medicine, Li Ka Shing Faculty of Medicine, The University of Hong Kong, Pok Fu Lam, Hong Kong Special Administrative Region, PR China; 2 Department of Microbiology, Li Ka Shing Faculty of Medicine, The University of Hong Kong, Pok Fu Lam, Hong Kong Special Administrative Region, PR China

## Abstract

Avian influenza virus H9N2 isolates cause a mild influenza-like illness in humans. However, the pathogenesis of the H9N2 subtypes in human remains to be investigated. Using a human alveolar epithelial cell line A549 as host, we found that A/Quail/Hong Kong/G1/97 (H9N2/G1), which shares 6 viral "internal genes" with the lethal A/Hong Kong/156/97 (H5N1/97) virus, replicates efficiently whereas other H9N2 viruses, A/Duck/Hong Kong/Y280/97 (H9N2/Y280) and A/Chicken/Hong Kong/G9/97 (H9N2/G9), replicate poorly. Interestingly, we found that there is a difference in the translation of viral protein but not in the infectivity or transcription of viral genes of these H9N2 viruses in the infected cells. This difference may possibly be explained by H9N2/G1 being more efficient on viral protein production in specific cell types. These findings suggest that the H9N2/G1 virus like its counterpart H5N1/97 may be better adapted to the human host and replicates efficiently in human alveolar epithelial cells.

## Findings

Genetic characterization and phylogenetic analysis revealed that there are multiple lineages of H9N2 viruses isolated from various types of poultry including chickens, ducks, quail and pigeons. The H9N2 virus lineages found to be the most prevalent in poultry in southern China include the H9N2/G1-like lineage represented by A/Quail/Hong Kong/G1/97 (H9N2/G1) and the H9N2/Y280-like lineage represented by A/Duck/Hong Kong/Y280/97 (H9N2/Y280) and A/Chicken/Hong Kong/G9/97 (H9N2/G9) since 1997 [1]. These H9N2 lineages continued to disseminate in domestic poultry with the development of multiple reassortant subtypes from East Asia to the Middle East [2]. Additionally, avian-to-mammalian transmissions of H9N2 viruses were reported in Southeastern China [3].

H9N2 viruses have repeatedly infected humans albeit causing a mild disease [3-5]. The low pathogenic H9N2 virus is widespread in poultry across Asia and Europe with ample opportunities for interaction with humans. It has caused infection in pigs (a putative mixing vessel for pandemic emergence) and causes severe disease in experimentally infected mice without prior adaptation [6]. The virus has an affinity for binding sialic acid receptors found on the human upper respiratory tract [7]. As past pandemics were not caused by highly pathogenic avian influenza viruses, the endemic of H9N2 viruses in poultry as well as their tropism for humans are at least as likely to cause the potential pandemic as the H5N1 virus, which is still the focus of attention [8]. Additionally, the H9N2/G1 viruses share six viral genes (viz. PB2, PB1, PA, NP, M and NS) with the lethal H5N1 viruses causing human disease in 1997 [1]. Furthermore, an H9N2 avian-human reassortant virus has been shown to have enhanced replication and efficient transmission in ferrets [9]. Thus H9N2 virus group is regarded by the World Health Organization as a potential pandemic candidate. Therefore we examined the replication characteristics of H9N2 virus lineages in the human lung epithelial cell line (A549) so that we may obtain an insight into the pathogenesis of H9N2 viruses in humans.

**Figure 1:**
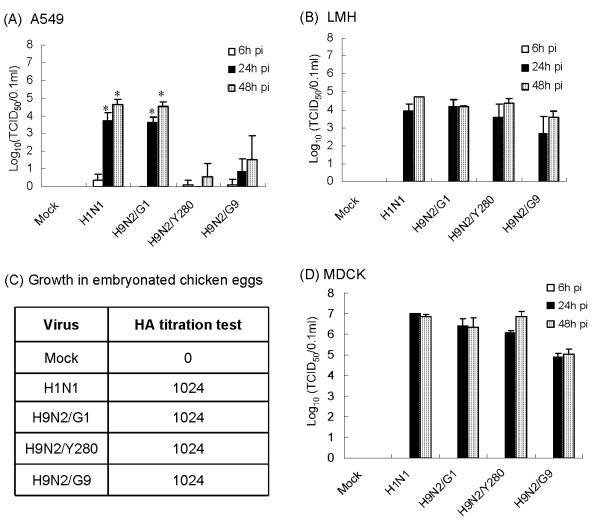
**Replication of H9N2 subtypes in different cell types and chicken embryonated eggs**. (A) A549, (B) LMH and (D) MDCK cells were infected by H1N1, H9N2/G1, H9N2/Y280 or H9N2/G9 at an m.o.i. of 0.01, and (C) chicken eggs were infected with the viruses at TCID_50 _of 2 × 10^6^. Viral titres of the infected cells were measured at 6 h, 24 h and 48 h post-infection (pi). Each point represents the mean of viral titers for three independent experiments and the titres were statistically analyzed by the two tailed, paired t-test; *:p < 0.05; Mock, uninfected cells.

To examine the replication efficiency of the H9N2 virus in human cells, A549 cells were infected with H9N2/G1, H9N2/Y280 or H9N2/G9 at a multiplicity of infection (m.o.i.) of 0.01 [10-12]. The culture supernatants were collected at 6 h, 24 h and 48 h post-infection and the viral titres were determined by tissue culture infectious dose (TCID_50_) assays using Madin-Darby canine kidney (MDCK) cells. Their replication efficiencies were compared with the human influenza A/Hong Kong/54/98 virus (H1N1). As shown in Fig. 1A, the TCID_50 _titre of H9N2/G1 viruses at 24 h and 48 h post-infection were 10^3.6 ^and 10^4.5 ^per 0.1 ml, respectively. Similar viral titres were observed in H1N1-infected cells at the corresponding time points. In contrast, both H9N2/Y280 and H9N2/G9 viruses exhibited poor replication competence in A549 cells (Fig 1A). Thus our results showed that H9N2/G1 viruses replicated as efficiently as H1N1 in the human lung epithelial A549 cells.

Next we examine the host-specific effects on the replication of H9N2 viruses, the leghorn male hepatoma chicken liver (LMH) cells were infected by H9N2 subtypes or H1N1 virus. We also infected embryonated chicken eggs with these viruses and their viral titres were determined by a hemagglutination assay. As shown in Fig. 1B and 1C, the three H9N2 viruses and H1N1 virus replicated to similar levels in LMH cells and embryonated chicken eggs. Furthermore, we found that all of the influenza viruses replicated well in MDCK cells (Fig. 1D). Hence, our results showed that all H9N2 subtypes tested replicated efficiently in MDCK, LMH cells and embryonated chicken eggs.

**Figure 2:**
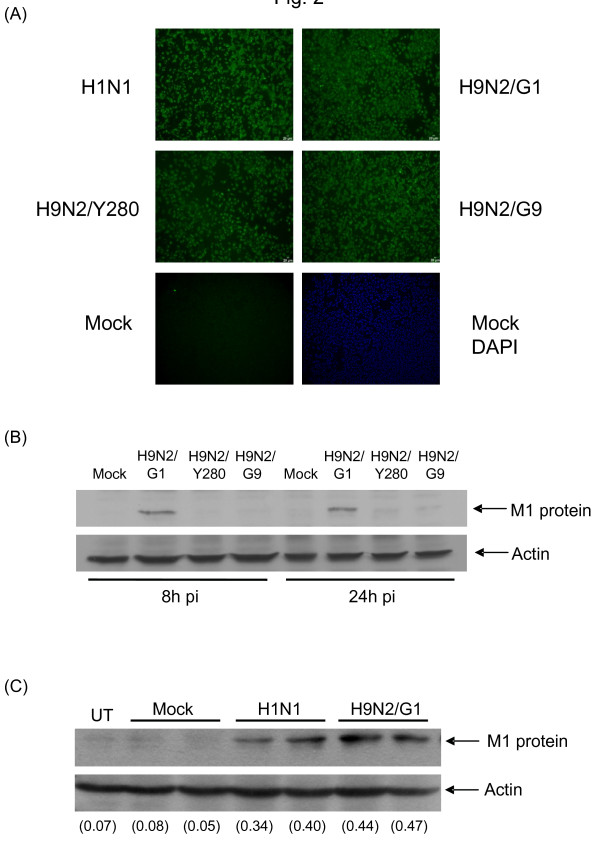
**Expression of viral nucleoprotein and matrix protein in A549 cells**. A549 cells were infected with H1N1, H9N2/G1, H9N2/Y280 or H9N2/G9 at an m.o.i. of 2 and cultured with DMEM without adding N-Tosyl-L-phenylalaninechloromethyl ketone-treated trypsin. (A) The infected cells were stained with FITC-conjugated monoclonal antibody specific for influenza nucleoproteins at 24 h post-infection (pi) and were visualized by fluorescence microscopy. Mock-treated cells (at the lower right corner) were counter-stained with DAPI. (B) A549 cells were infected with H9N2/G1, H9N2/Y280 or H9N2/G9 at an m.o.i. of 2 or mock treated. Total proteins were harvested at 8 h or 24 h post-infection (pi) and M1 proteins of influenza A were examined by Western analysis. (C) A549 cells were infected with influenza H1N1 or H9N2/G1 viruses at an m.o.i. of 2 or mock treated. Total proteins were harvested at 8 h or 24 h pi and M1 proteins of influenza A were examined by Western analysis. Equal loading of protein samples was determined with anti-actin antibodies. The densities of the protein bands were determined by using Bio-Rad Quantity One imaging software. The values in the parenthesis are relative M1 protein intensities compared with those of the corresponding actin. Mock, uninfected cells; M1, matrix protein; UT, untreated; DAPI, 4'-6-Diamidino-2-phenylindole.

**Figure 3:**
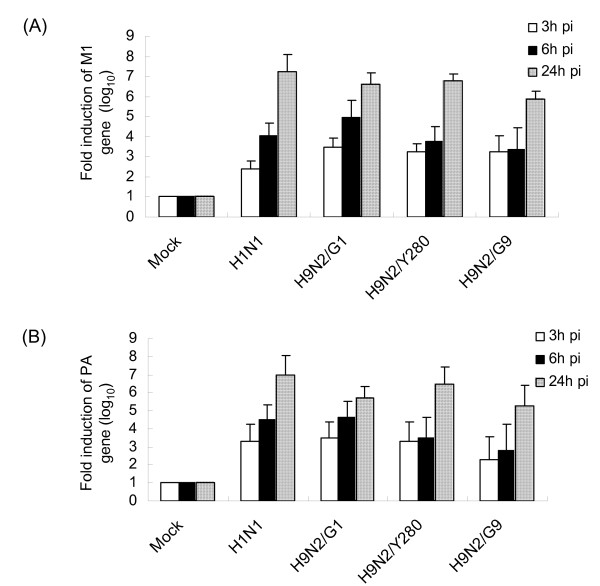
**Quantitative analysis of the RNA levels of M1 and PA in A549 cells**. (A) A549 cells were infected with H9N2/G1, H9N2/Y280 or H9N2/G9 at an m.o.i. of 2 or mock treated. Total RNA samples of the virus-infected cells were collected at 3 h, 6 h and 24 h post-infection (pi) and assayed by quantitative RT-PCR. The mRNA levels of (A) M1 and (B) PA, normalized to β-actin gene, were compared to the uninfected samples. Mock, uninfected cells; M1, matrix protein; PA, acid polymerase protein.

To delineate the mechanisms underlying the differences in replication of H9N2 subtypes, we investigated the infectivity, transcription and translation of different viruses in A549 cells. A549 cells were first infected by H9N2/G1, H9N2/Y280, H9N2/G9 or H1N1 at an m.o.i. of 2 and the expression of viral nucleoprotein (NP) and matrix protein (M1) in the infected cells was examined by using immunofluorescent staining and Western analysis, respectively. Positive staining of the NP protein was found in both H9N2-infected and H1N1-infected A549 cells at 24 h post-infection (Fig. 2A). Interestingly, strong M1 protein expression was observed in cells infected with H9N2/G1 at 8 h and 24 h post-infection by using antibody against M1 protein of influenza type A viruses but not those infected with H9N2/Y280 or H9N2/G9 virus (Fig 2B). To exclude the possibility of the specificity of the antibody, we compared the expression of M1 protein in A549 cells infected with H1N1 or H9N2/G1 virus. Results similar to the viral titres, there were no significant differences of M1 protein expression in these two viruses (Fig. 2C). Next, we measured the mRNA level of M1 protein and acid polymerase protein (PA) in virus-infected A549 cells at 3 h, 6 h and 24 h post-infection by using quantitative RT-PCR assay [11]. The coding regions of the respective viral genes in H9N2 and H1N1 viruses were amplified by the conserved gene-specific PCR primers. Both M1 and PA mRNA were detected at 3 h post-infection and their levels increased at 24 h post-infection in H9N2- or H1N1-infected cells (Fig. 3A, B). We found that there was no significant difference in the level of the viral mRNA in the cells infected by either virus subtypes. Taken together, our results showed that the virus entry of the examined H9N2 subtypes seems comparable but there is a difference in M1 protein expression in human lung epithelial A549 cells. However, the discrepancy of the mRNA synthesis and protein expression in A549 cells with H9N2/Y280 or H9N2/G9 infection remains to be investigated.

Since 1999, the H9N2 viruses have been intermittently isolated from patients manifesting influenza-like symptoms [3,4] and these viruses pose a pandemic threat. However, the pathogenicity of the avian influenza H9N2 viruses remains to be investigated. Recent studies have shown that avian or human influenza viruses including H5N1, H7N1 and H1N1 can infect cells in the human lung epithelium [13,14]. In addition, a previous report showed that avian H5N1 virus can replicate both in the upper and lower respiratory tract [15]. These findings imply that avian influenza virus other than H5N1 may have similar replicating ability in human cells and tissues.

In this report, we found that all three H9N2 lineages tested can infect different cell types including A549 cells, MDCK and LMH as evident by the detection of H9N2 viral protein NP in the infected cells and embryonated chicken eggs. Interestingly, only H9N2/G1 virus could produce a high titre of virus particles in A549 cells comparable to the H1N1 infection. These results suggested that the disease severity of H9N2/G1-infected patients may be associated with the replication ability of the virus in specific cell types. From our results, we showed that the viral growth efficiency may not be due to the difference of infectivity of the virus subtypes. Instead, we found that the translation of M1 protein is impaired in H9N2/Y280 and H9N2/G9. Previous reports have shown that M1 forms the major structural component of the virion and plays an important role in virus budding and assembly [16,17]. However, the detailed mechanisms underlying the translational efficiency of H9N2/G1 virus and its differences from that of the less efficient H9N2/Y280 and H9N2/G9 viruses in A549 cells remain to be investigated. Enami et al. previously showed that the NS1 can stimulate the translation of the M1 protein [18]. As the NS1 of H9N2/G1 is highly conserved to the human pathogenic H5N1 virus which were found during 1997, it is possible that the NS1 of H9N2/G1 may lead to a high translational efficiency on M1 protein production in human host while the other H9N2 subtypes are unable to do so.

The genetic background of different H9N2 subtypes (Table 1) may contribute to the differences in their respective replication ability. In regard to the hemagglutinin (HA) gene, both H9N2/Y280 and H9N2/G9 viruses belong to the same HA sublineage of H9N2 viruses, while H9N2/G1 and H5N1/97 viruses belong to a different sublineage [19]. Moreover, H9N2/G1 and H5N1/97 viruses possess the similar replication complexes that are pathogenic in mice [19]. The H9N2/G1 viruses share six internal genes, including PB2 with the H5N1/97 viruses, while the H9N2/G9 lineage viruses share PB1 and PB2 genes. By contrast, the H9N2/Y280 virus does not have this share features in its viral genome [1]. It has been shown by other reports that the PB2 protein in H5N1 viruses contributes to the host adaptation and virus growth in humans [20,21]. Furthermore, it has been demonstrated that H5N1/97 viruses replicate efficiently in primary alveolar epithelial cells and the viral titre of the infected cells is comparable to that of the human influenza H1N1 viruses [22]. However, it is notable that the internal gene complements of the current Z genotype H5N1 viruses are different to those of H5N1/97 and H9N2/G1 [23]. Thus, the adaptation of H5N1 to grow in human cells reflects its potential ability to cross the species restriction resulting in its occasional transmission from the avian hosts to human.

**Table 1:** Segments comparison between different H9N2 and H5N1 subtypes

	Source of the segments							
	
Virus	HA	NA	NP	M	NS	PA	PB1	PB2
A/Quail/HongKong/G1/97 (H9N2/G1)	G1	G1	G1	G1	G1	G1	G1	G1
A/Duck/HongKong/Y280/97 (H9N2/Y280)	Y280	Y280	Y280	Y280	Y280	Y280	Y280	Y280
A/Chicken/HongKong/G9/97 (H9N2/G9)	Y280	Y280	Y280	Y280	Y280	Y280	G1	G1
A/HongKong/156/97 (H5N1/97)	H5*	H6^#^	G1	G1	G1	G1	G1	G1
HongKong/1073/99 (Human isolate H9N2/99)	G1	G1	G1	G1	G1	G1	G1	G1
HongKong/2108/03 (Human isolate H9N2/03)	Y280	G9^##^	Y280	G1	Y280	H5**	G1	G1

NB. *A/Goose/Guangdong/1/96, **H5N1/01-like, ^#^A/Teal/HongKong/W312/97,

^##^A/Chicken/HongKong/G9/97

Since both of the H9N2 human isolates from 1999 and 2003 caused mild respiratory symptoms in infected patients, we postulate that there may be some common factors that lead to effective infection among those viruses. The HA, NA, NP genes of H9N2 viruses that have infected humans belonged to either H9N2/G1 or H9N2 Y280-like sub-lineages. Interestingly however, irrespective of the derivation of the HA and NA genes, the PB2, PB1 and M genes of these human isolates all belong to the H9N2/G1 sublineage. The PA genes of these viruses belong to either the H9N2/G1 or the contemporary Z genotype H5N1 viruses [4]. It is notable that the polymerase gene complex and the M gene of the viruses arise either from the H9N2/G1 - H5N1/97 sublineage or from the recent H5N1-Z genotype lineage associated with recent human H5N1 disease but not from the H9N2/Y280-sublineage. Since H9N2/G9 viruses which share the H9N2/G1-like PB1 and PB2 genes also fail to replicate in the human alveolar epithelial A549 cells, it would possibly implicate the need for all three polymerase gene segments and/or the M gene segment for efficient replication in human cells.

Infection of patients with avian influenza virus subtypes including H4N8, H6N1, and H10N7 is inefficient and this may be due to the inefficient virus replication competence in human cells [24]. Here, we demonstrated the differential replication of H9N2 virus subtypes in human lung epithelial cells. It provides a cellular model to investigate the mechanisms of replication of avian influenza viruses in humans, as well as the interaction of viral proteins with host factors, which may contribute to the pathogenesis of avian influenza virus.

## Competing interests

The authors declare that they have no competing interests.

## Authors' contributions

DCWL participated in experimental design, data analysis and manuscript preparation, CMKP performed experiments, data analysis and draft the manuscript, AHYL carried out experiments and data analysis, MP participated in data analysis and manuscript preparation, ASYL participated in project design, data analysis and manuscript preparation. All authors read and approved the final manuscript.

## References

[B1] Guan Y Shortridge KF Krauss S Webster RG Molecular characterization of H9N2 influenza viruses: were they the donors of the "internal" genes of H5N1 viruses in Hong Kong? Proc Natl Acad Sci USA 1999 96 9363 9367 10.1073/pnas.96.16.9363 10430948 PMC17788

[B2] Xu KM Smith GJ Bahl J Duan L Tai H Vijaykrishna D Wang J Zhang JX Li KS Fan XH Webster RG Chen H Peiris JS Guan Y The genesis and evolution of H9N2 influenza viruses in poultry from southern China, 2000 to 2005 J Virol 2007 81 10389 10401 10.1128/JVI.00979-07 17652402 PMC2045440

[B3] Peiris M Yuen KY Leung CW Chan KH Ip PL Lai RW Orr WK Shortridge KF Human infection with influenza H9N2 Lancet 1999 354 916 917 10.1016/S0140-6736(99)03311-5 10489954

[B4] Butt KM Smith GJ Chen H Zhang LJ Leung YH Xu KM Lim W Webster RG Yuen KY Peiris JS Guan Y Human infection with an avian H9N2 influenza A virus in Hong Kong in 2003 J Clin Microbiol 2005 43 5760 5767 10.1128/JCM.43.11.5760-5767.2005 16272514 PMC1287799

[B5] Lin YP Shaw M Gregory V Cameron K Lim W Klimov A Subbarao K Guan Y Krauss S Shortridge K Webster R Cox N Hay A Avian-to-human transmission of H9N2 subtype influenza A viruses: relationship between H9N2 and H5N1 human isolates Proc Natl Acad Sci USA 2000 97 9654 9658 10.1073/pnas.160270697 10920197 PMC16920

[B6] Hossain MJ Hickman D Perez DR Evidence of expanded host range and mammalian-associated genetic changes in a duck H9N2 influenza virus following adaptation in quail and chickens PLoS ONE 2008 3 e3170 10.1371/journal.pone.0003170 18779858 PMC2525835

[B7] Matrosovich MN Krauss S Webster RG H9N2 influenza A viruses from poultry in Asia have human virus-like receptor specificity Virology 2001 281 156 162 10.1006/viro.2000.0799 11277689

[B8] Peiris JS de Jong MD Guan Y Avian influenza virus (H5N1): a threat to human health Clin Microbiol Rev 2007 20 243 267 10.1128/CMR.00037-06 17428885 PMC1865597

[B9] Wan H Sorrell EM Song H Hossain MJ Ramirez-Nieto G Monne I Stevens J Cattoli G Capua I Chen LM Donis RO Busch J Paulson JC Brockwell C Webby R Blanco J Al-Natour MQ Perez DR Replication and transmission of H9N2 influenza viruses in ferrets: evaluation of pandemic potential PLoS ONE 2008 3 e2923 10.1371/journal.pone.0002923 18698430 PMC2500216

[B10] Cheung CY Poon LL Lau AS Luk W Lau YL Shortridge KF Gordon S Guan Y Peiris JS Induction of proinflammatory cytokines in human macrophages by influenza A (H5N1) viruses: a mechanism for the unusual severity of human disease? Lancet 2002 360 1831 1837 10.1016/S0140-6736(02)11772-7 12480361

[B11] Lee DC Cheung CY Law AH Mok CK Peiris M Lau AS p38 mitogen-activated protein kinase-dependent hyperinduction of tumor necrosis factor alpha expression in response to avian influenza virus H5N1 J Virol 2005 79 10147 10154 10.1128/JVI.79.16.10147-10154.2005 16051807 PMC1182678

[B12] Mok CK Lee DC Cheung CY Peiris M Lau AS Differential onset of apoptosis in influenza A virus H5N1- and H1N1-infected human blood macrophages J Gen Virol 2007 88 1275 1280 10.1099/vir.0.82423-0 17374772

[B13] Kogure T Suzuki T Takahashi T Miyamoto D Hidari KI Guo CT Ito T Kawaoka Y Suzuki Y Human trachea primary epithelial cells express both sialyl(alpha2-3)Gal receptor for human parainfluenza virus type 1 and avian influenza viruses, and sialyl(alpha2-6)Gal receptor for human influenza viruses Glycoconj J 2006 23 101 106 10.1007/s10719-006-5442-z 16575527

[B14] Matrosovich MN Matrosovich TY Gray T Roberts NA Klenk HD Human and avian influenza viruses target different cell types in cultures of human airway epithelium Proc Natl Acad Sci USA 2004 101 4620 4624 10.1073/pnas.0308001101 15070767 PMC384796

[B15] Nicholls JM Chan MC Chan WY Wong HK Cheung CY Kwong DL Wong MP Chui WH Poon LL Tsao SW Guan Y Peiris JS Tropism of avian influenza A (H5N1) in the upper and lower respiratory tract Nat Med 2007 13 147 149 10.1038/nm1529 17206149

[B16] Gomez-Puertas P Albo C Perez-Pastrana E Vivo A Portela A Influenza virus matrix protein is the major driving force in virus budding J Virol 2000 74 11538 11547 10.1128/JVI.74.24.11538-11547.2000 11090151 PMC112434

[B17] Nayak DP Hui EK Barman S Assembly and budding of influenza virus Virus Res 2004 106 147 165 10.1016/j.virusres.2004.08.012 15567494 PMC7172797

[B18] Enami K Sato TA Nakada S Enami M Influenza virus NS1 protein stimulates translation of the M1 protein J Virol 1994 68 1432 1437 7508995 10.1128/jvi.68.3.1432-1437.1994 PMC236597

[B19] Guan Y Shortridge KF Krauss S Chin PS Dyrting KC Ellis TM Webster RG Peiris M H9N2 influenza viruses possessing H5N1-like internal genomes continue to circulate in poultry in southeastern China J Virol 2000 74 9372 9380 10.1128/JVI.74.20.9372-9380.2000 11000205 PMC112365

[B20] Hatta M Gao P Halfmann P Kawaoka Y Molecular basis for high virulence of Hong Kong H5N1 influenza A viruses Science 2001 293 1840 1842 10.1126/science.1062882 11546875

[B21] Shinya K Hamm S Hatta M Ito H Ito T Kawaoka Y PB2 amino acid at position 627 affects replicative efficiency, but not cell tropism, of Hong Kong H5N1 influenza A viruses in mice Virology 2004 320 258 266 10.1016/j.virol.2003.11.030 15016548

[B22] Chan MC Cheung CY Chui WH Tsao SW Nicholls JM Chan YO Chan RW Long HT Poon LL Guan Y Peiris JS Proinflammatory cytokine responses induced by influenza A (H5N1) viruses in primary human alveolar and bronchial epithelial cells Respir Res 2005 6 135 10.1186/1465-9921-6-135 16283933 PMC1318487

[B23] Li KS Guan Y Wang J Smith GJ Xu KM Duan L Rahardjo AP Puthavathana P Buranathai C Nguyen TD Estoepangestie AT Chaisingh A Auewarakul P Long HT Hanh NT Webby RJ Poon LL Chen H Shortridge KF Yuen KY Webster RG Peiris JS Genesis of a highly pathogenic and potentially pandemic H5N1 influenza virus in eastern Asia Nature 2004 430 209 213 10.1038/nature02746 15241415

[B24] Beare AS Webster RG Replication of avian influenza viruses in humans Arch Virol 1991 119 37 42 10.1007/BF01314321 1863223

